# Crosstalk between Cancer Cells and Fibroblasts for the Production of Monocyte Chemoattractant Protein-1 in the Murine 4T1 Breast Cancer

**DOI:** 10.3390/cimb43030122

**Published:** 2021-10-22

**Authors:** Mayu Imamura, Tiantian Li, Chunning Li, Masayoshi Fujisawa, Naofumi Mukaida, Akihiro Matsukawa, Teizo Yoshimura

**Affiliations:** 1 Department of Pathology and Experimental Medicine, Graduate School of Medicine, Dentistry and Pharmaceutical Sciences, Okayama University, 2-5-1 Shikata, Kita-ku, Okayama 700-8558, Japan; ptnm7qkv@s.okayama-u.ac.jp (M.I.); opheliali1994tiantian@gmail.com (T.L.); 18640512230@163.com (C.L.); mfujisawa@okayama-u.ac.jp (M.F.); amatsu@md.okayama-u.ac.jp (A.M.); 2 Division of Molecular Bioregulation, Cancer Research Institute, Kanazawa University, Kakuma-machi, Kanazawa 920-1192, Japan; mnao.otcyt@gmail.com

**Keywords:** breast cancer, chemokine, lung metastasis, fibroblasts, macrophages, tumor microenvironment

## Abstract

The chemokine monocyte chemoattractant protein-1 (MCP-1/CCL2) is shown to promote the progression of breast cancer. We previously identified cancer cell-derived granulocyte-macrophage colony-stimulating factor (GM-CSF) as a potential regulator of MCP-1 production in the murine 4T1 breast cancer, but it played a minimum role in overall MCP-1 production. Here, we evaluated the crosstalk between 4T1 cells and fibroblasts. When fibroblasts were co-cultured with 4T1 cells or stimulated with the culture supernatants of 4T1 cells (4T1-sup), MCP-1 production by fibroblasts markedly increased. 4T1 cells expressed mRNA for platelet-derived growth factor (PDGF)-a, b and c, and the PDGF receptor inhibitor crenolanib almost completely inhibited 4T1-sup-induced MCP-1 production by fibroblasts. However, PDGF receptor antagonists failed to reduce MCP-1 production in tumor-bearing mice. Histologically, 4T1 tumors contained a small number of αSMA-positive fibroblasts, and Mcp-1 mRNA was mainly associated with macrophages, especially those surrounding necrotic lesions on day 14, by in situ hybridization. Thus, although cancer cells have the capacity to crosstalk with fibroblasts via PDGFs, this crosstalk does not play a major role in MCP-1 production or cancer progression in this model. Unraveling complex crosstalk between cancer cells and stromal cells will help us identify new targets to help treat breast cancer patients.

## 1. Introduction

Breast cancer is the most common cancer in women, and the majority of breast cancer-related deaths are caused by metastasis [1]. The lung, brain, bone and liver are the preferential sites of breast cancer metastasis. Lung metastasis is particularly concerning because it is associated significantly with patients’ morbidity and mortality rate. Current therapeutic methods for metastatic breast cancer are ineffective, and there is no reliable method to predict the development of metastatic lesions. A better understanding of the mechanisms that drive breast cancer metastasis is crucial to identifying novel biomarkers and therapeutic targets for breast cancer [1].

Lung metastasis of breast cancer cells involves several processes and is regulated by mediators secreted in primary tumors [1]. The murine 4T1 breast cancer is a triple-negative (TN) subtype of breast cancer and a subclone of the original mammary tumor that arose in a BALB/c mouse foster-nursed by a C3H female mouse [2,3]. BALB/c mice injected with 4T1 cells into mammary pads spontaneously develop lung metastases, similar to TN breast cancer patients [4]. Unlike other transplantable cancer models, 4T1 cells consist of heterogenous clonal subpopulations with different morphology, behaviors and gene expression profiles [5]. These unique features of 4T1 cells make them a terrific model to analyze the complex mechanisms involved in the lung metastasis of breast cancer cells.

The chemokine monocyte chemoattractant protein-1 (MCP-1)/CCL2 is a potent chemoattractant for monocytes and has been shown to play an important role in the recruitment of immunosuppressive, proangiogenic tumor-associate macrophages (TAMs) and the promotion of lung metastasis of breast cancer cells [6,7]. In both human breast cancer patients [8,9,10,11] and a murine breast cancer model [12], non-tumor stromal cells are important sources of MCP-1. To develop potential therapeutic modalities to inhibit MCP-1 production in tumor microenvironments (TMEs), we previously analyzed the crosstalk between 4T1 breast cancer cells and macrophages and identified cancer cell-derived granulocyte-macrophage colony-stimulating factor (GM-CSF/CSF2) as a potential key molecule that regulates high-level MCP-1 production in breast cancer TMEs. However, neutralization of GM-CSF or deletion of the *Csf2* gene in cancer cells had little effect on the overall MCP-1 production in tumor-bearing mice, indicating the presence of additional mechanisms that induce MCP-1 production in TMEs [13].

Fibroblasts are a major component of tumor stroma, and those activated by growth factors and associated with cancer are termed cancer-associated fibroblasts (CAFs). CAFs express several markers, including α-smooth muscle actin (αSMA; also known as actin alpha 2), fibroblast activation protein (FAP) and PDGF receptor-α (PDGFRα) and PDGFRβ. Activation by growth factors, such as transforming growth factor-β (TGF-β), PDGFs and fibroblast growth factor-2, results in the production of cytokines and chemokines important for cancer progression [14], leading to the hypothesis that fibroblasts activated by a product(s) of 4T1 cells significantly contribute to the overall MCP-1 production in 4T1 tumors.

In the present study, we examined the presence of crosstalk between cancer cells and fibroblasts that could contribute to the production of MCP-1 in the 4T1 TME both in vitro and in vivo. For in vivo experiments, we focused on the early phase of tumor development (1 to 2 weeks after cancer cell inoculation) when MCP-1 production in tumors appeared most active and metastatic foci in the lung were initially established. We first identified in vitro that PDGFs released by 4T1 cells were potent inducers of MCP-1 production by fibroblasts and that an inhibitor of PDGFRs almost completely inhibited 4T1 cell-induced MCP-1 production by fibroblasts. However, inhibition of PDGFRs had no effect in vivo. Histologically, 4T1 tumors contained only a small number of activated fibroblasts, and *Mcp-1* mRNA was mostly associated with macrophages by in situ hybridization (ISH), likely contributing to the lack of reduction in MCP-1 production by PDGFR antagonists. Better understanding the mechanisms of interactions between cancer cells and stromal cells is crucial to identifying new molecular targets and to develop new cancer treatments.

## 2. Materials and Methods

### 2.1. Reagents

RPMI-1640 medium was purchased from Sigma-Aldrich (St. Louis, MO, USA) and Nakarai Tesque, Kyoto, Japan. Dulbecco’s modified Eagle’s medium (DMEM) with high glucose was from Nakarai Tesque. Phosphate buffered saline (PBS) and citric acid were from Wako Pure Chemical Corp, Osaka, Japan. TRIsure^®^ reagent was from Nippon Genetics, Tokyo, Japan. Fetal bovine serum (FBS) was from HyClone, Logan, UT, USA and Gibco, Grand Island, NY, USA. L-glutamine, penicillin/streptomycin, trypsin-EDTA, sodium pyruvate solution and RNAlater were from Life Technologies, Gaithersburg, MD, USA. High Pure RNA Isolation Kit was from Roche, Mannheim, Germany. BCA protein assay kit was from Takara, Tokyo, Japan. A mouse monoclonal Ab against human PDGF-A (clone E-10) was from Santa Cruz, Dallas, TX, USA. Anti-Ly6G rat monoclonal IgG (clone 1A8), recombinant mouse PDGF-AA and BB were from BioLegend, San Diego, CA, USA. Anti-F4/80 rat monoclonal IgG (clone BM8) was from eBioscience, San Diego, CA, USA. A rabbit polyclonal Ab against αSMA was from Abcam, Cambridge, MA, USA. Trapidil and crenolanib were from Selleck Chemicals, Osaka, Japan.

### 2.2. 4T1 Cells

Murine 4T1 breast cancer 4T1 cells were obtained from (ATCC, Manassas, VA, USA) and grown in RPMI 1640 supplemented by 10% FBS, 100 μM glutamine, 100 U/mL penicillin, 100 μg/mL streptomycin and 1 mM sodium pyruvate (complete medium), as previously described [12]. The generation of GM-CSF-deficient 4T1 cells (A8) was previously reported [13]. To generate 4T1 cell culture supernatant (4T1-sup), 4T1 cells were grown in the complete medium in T-75 tissue culture flasks. When the color of the media turned slightly yellow, cell-free supernatants were collected by centrifugation at 1200 rpm for 10 min. Cell-free supernatants were concentrated by using Amicon Ultra-15 Centrifuge filters (Merck Millipore Ltd., Cork, Ireland).

### 2.3. Fibroblasts

NIH/3T3 cells (Japanese Cancer Research Resources Bank, Osaka, Japan) were grown in DMEM supplemented by 10% FBS, 100 μM glutamine, 100 U/mL penicillin and 100 μg/mL streptomycin. Lung fibroblasts were isolated from normal BALB/c mice, as previously described [15,16]. In brief, lungs were isolated under sterile conditions, minced into small fragments (1 mm^3^), placed on tissue culture plates containing high glucose DMEM with 15% FBS and incubated at 37 °C in a 15% CO_2_ atmosphere for 5 days. Lung tissues were removed from the plates, and fibroblasts were grown to 80% confluence and then transferred to T-75 flasks. Fibroblasts were harvested by trypsin-EDTA and used.

### 2.4. Activation of Fibroblasts

For co-culture experiments, 1 × 10^4^ 3T3 cells were seeded in 24-well plates with different numbers of 4T1 cells. After overnight incubation at 37 °C, the medium was replaced by 0.5 mL fresh complete medium, and cells were incubated for an additional 4 days at 37 °C. Cell-free culture supernatants were collected and stored at −30 °C until use. For stimulation of 3T3 cells with 4T1-sup, 5 × 10^4^ 3T3 cells were seeded into 24-well plates. After overnight incubation at 37 °C, the medium was replaced by 0.5 mL fresh medium containing different concentrations of 4T1-sup and the cells were incubated for 24 h at 37 °C. Cell-free culture supernatants were collected and stored at −30 °C until use. For stimulation of normal mouse fibroblasts, 5 × 10^4^ fibroblasts were seeded into 24-well plates. After overnight incubation at 37 °C, the medium was replaced by a fresh medium containing different concentrations of 4T1-sup. After a 24-h incubation, cell-free culture supernatants were collected and stored at −30 °C until use.

To evaluate the effects of PDGFs on fibroblasts, 5 × 10^4^ 3T3 cells were seeded into 24-well plates. After overnight incubation at 37 °C, the medium was replaced by 0.5 mL fresh medium containing recombinant PDGF-AA or PDGF-BB and the cells were incubated for 24 h at 37 °C. Cell-free culture supernatants were collected and stored at −30 °C until use.

To block PDGFR, 5 × 10^4^ 3T3 cells were seeded into 24-well plates. After overnight incubation at 37 °C, the medium was replaced by 0. 5 mL fresh medium containing DMSO or 0.2 or 2 μM crenolanib. After a 30-min incubation at 37 °C, the same volume of medium or 4T1-sup was added, and the cells were incubated for an additional 24 h. Cell-free culture supernatants were collected and stored at −30℃ until use. Total RNA was extracted 1 h after stimulation and stored at −80 °C until use.

### 2.5. Transplantation of 4T1 Cells

Wild-type female BALB/c mice were purchased from Japan SLC, Inc., Hamamatsu, Japan, MD. One million 4T1 cells or A8 cells were grown to 50% to 80% confluence in T-75 tissue culture flasks in RPMI 1640 supplemented with 10% FBS, 2 mM L-glutamine, penicillin/streptomycin and sodium pyruvate. Cells were detached with 0.2% trypsin-EDTA, washed once with medium, three times with PBS and resuspended in PBS at 1 × 10^6^/mL. One hundred μL of cell suspension (1 × 10^5^ cells) was injected into the left 3rd mammary pad of female mice. Blood was collected by heart puncture, and sera were isolated and stored at −80 °C until use. The tumors were harvested, and a half was fixed in 10% neutral buffered formalin (Wako) and the other half was in RNAlater. Tumor length and width were measured using a caliper, and tumor volume was calculated using the following formula: Volume = (width) 2 × length/2.

To block PDGFR, 20 mg/kg of trapidil dissolved in PBS or PBS was intraperitoneally (i.p.) injected twice a day for 7 days from day 5. One hundred μg/kg of crenolanib in 0.5 mL PBS or 0.5 mL of 0.1% DMSO in PBS was also i.p. injected twice a day for 3 days from day 5. Twelve hours after the final injection, mice were euthanized. In another experiment, 0.1 mL of 10 μM crenolanib in PBS was intratumorally (i.t.) injected 3 or 5 times every 12 h from day 5, and the mice were euthanized 12 h after the final injection.

### 2.6. Activation of Macrophages by Necrotic Cells

Mouse peritoneal exudate cells (PEC) were induced by intraperitoneal injection of 3% thioglycollate and harvested 3–4 days later by peritoneal lavage using 5 mL cold PBS. After centrifugation, peritoneal exudate cells were resuspended in RPMI-1640 containing 10% FBS, penicillin and streptomycin at the concentration of 1 × 10^6^ and then cultured for 24 h in the presence or absence of stimuli. Ten million 4T1 or A8 cells were rinsed with PBS twice, resuspended in 1.5 mL PBS and then kept at −80 °C for 6 h. The frozen cells were thawed and used to stimulate macrophages.

### 2.7. Quantification of MCP-1 Concentration

The concentration of MCP-1 was measured using an ELISA kit specific for mouse MCP-1 (BioLegend) or by sandwich ELISA using anti-mouse MCP-1 antibodies (R&D Systems, Minneapolis, MN, USA). All samples were assayed in duplicate.

### 2.8. Western Blotting

4T1 cells were lysed in a lysis buffer (Cell Signaling, Danvers, MA, USA), briefly sonicated, incubated on ice for 30 min and then centrifuged at 12,000× *g* for 10 min. The protein concentrations were measured by BCA protein assay. Ten µg of cell lysate was loaded on a precast polyacrylamide gel (Thermo-Fisher, Carlsbad, CA, USA), and the proteins were fractionated by sodium dodecyl sulfate-polyacrylamide gel electrophoresis (Life Technologies) and transferred onto nitrocellulose membranes. After overnight incubation with a primary antibody, the membrane was washed and incubated with horseradish peroxidase-conjugated rabbit anti-mouse IgG (Santa Cruz Biotechnology, Santa Cruz, CA, USA). The presence of the protein of interest was visualized and quantitated with C-DiGit Blot scanner (Scrum, Tokyo, Japan).

### 2.9. qRT-PCR

The expression of the *Mcp-1/Ccl2 or Kc/Cxcl1* gene was assessed by qRT-PCR. Total RNA was extracted from non-activated or activated 3T3 cells by using TRIsure or High Pure RNA Isolation Kit. The quality and yield were assessed by Nanodrop spectrophotometry, and then cDNA was synthesized using a High-Capacity cDNA Reverse Transcription Kit (Applied Biosystems, Foster City, CA, USA). qRT-PCR was performed using the StepOne Plus Real-Time PCR system (Life Technologies). The expression of the *Mcp-1, Kc* and *Gapdh* mRNA was analyzed in duplicate by Taqman gene expression assays (Applied Biosystems). The expression level of each gene was normalized to that of the *Gapdh* gene and presented as a fold change over the expression of the control gene. The values of control groups were assigned an arbitrary value of 1.

### 2.10. RT-PCR

The expression of the *Pdgf-a, b, c* and *d* genes was examined by RT-PCR. Total RNA was isolated by TRIsure^®^ reagent. Synthesis of cDNA was performed using High-Capacity cDNA Reverse Transcription Kits. The annealing temperature was 57 °C for PDGFa and 56 °C for PDGFb, c and d. The primer sequences were as follows: PDGFa forward, 5′-CAAGACCAGGACGGTCATTT-3′, PDGFa reverse, 5′-ACTTTGGCCACCTTGACACT-3′; PDGFb forward, 5′-GATCTCTCGGAACCTCATCG-3′, PDFGb reverse, 5′-GGCTTCTTTCGCACAATCTC-3′; PDGFc forward 5′-GTCCATACGGGAAGAGCTAAAG-3′, PDGFc reverse, 5′-TCTACACACACAGTCACATTCC-3′; PDGFd forward, 5′-TGCCAACCTCAGGAGAGATGA-3′, PDGFd reverse, 5′-GCTGCTTCCGGTTGGAAATC-3′.

### 2.11. Immunohistochemistry

The tumors were fixed overnight with buffered 10% formalin and embedded in paraffin. Serial sections of 4 μm thickness were prepared from formalin-fixed and paraffin-embedded blocks collected as described above. One section was stained with H&E, and others were used for immunohistochemistry for each block. Immunostaining was performed manually by a conventional method: briefly, sections were deparaffinized in xylene and rehydrated in a sequence of descending concentrations of ethanol. Endogenous peroxidase reactivity was blocked with 3% H_2_O_2_ for 10 min. For Ly6G staining, the sections were submerged in 0.1 M citrate buffer (pH 6.0) and microwaved (700 W) continuously for 15 min in a pressure cooker. For F4/80 staining, the sections were treated with 200 μg/mL proteinase K (Roche) for 5 min at room temperature. The sections were then incubated with a respective primary antibody for 1.5 h at room temperature. After washing, the sections were treated with Histofine Simple Stain^TM^ MAX PO (Nichirei, Japan) according to the manufacturer’s instructions. Finally, the sections were counterstained with hematoxylin, dehydrated and mounted. Images were acquired using an Olympus BX43 light microscope connected to a DP73 digital camera (Olympus, Tokyo, Japan).

### 2.12. In Situ Hybridization (ISH)

Spatial expression of the *Mcp-1/Ccl2*, *adhesion G protein-coupled receptor E1* (*Adgre1*, the gene coding for F4/80) or *αSMA* mRNA in 4T1 tumors was analyzed by the RNAScope^®^ 2.5 Duplex Detection Kit (ACD, Inc., Hayward, CA, USA). Briefly, tissue sections in 5-μm thickness were baked for 1 h at 60 °C, deparaffinized in xylene, followed by dehydration in an ethanol series. The tissue sections were then treated in the retrieval buffer maintained at a boiling temperature (98 °C) on a hot plate for 20 min, rinsed in deionized water and treated with protease at 40 °C for 45 min in a Dako StatSpin Hybridizer (Agilent, Santa Clara, CA, USA). The tissue sections were then hybridized at 40 °C in a hybridization buffer with probes (mouse *Mcp-1/Ccl2*, C1 probe, #311791; mouse *Adgre1*, C2 probe, #460651-C2; mouse *αSMA*, C2 probe, #) for 3 h. After the hybridization, the tissue sections were washed with a wash buffer three times at room temperature, and then the signals were amplified. Chromogenic detection was performed using FastRed (*Adgre1* or *αSMA*) or FastGreen (*Mcp-1/Ccl2*), followed by counterstaining with hematoxylin. Photos were taken using an Olympus BX43 light microscope connected to a DP73 digital camera (Olympus, Tokyo, Japan).

### 2.13. Statistical Analysis

Statistical analysis was performed by Student’s t-test using the GraphPad Prism 9 (GraphPad Software, San Diego, CA, USA). A value of *p* < 0.05 was considered statistically significant.

## 3. Results

### 3.1. A Product(s) of 4T1 Cells Induces MCP-1 Production by Fibroblasts

**Figure 1:**
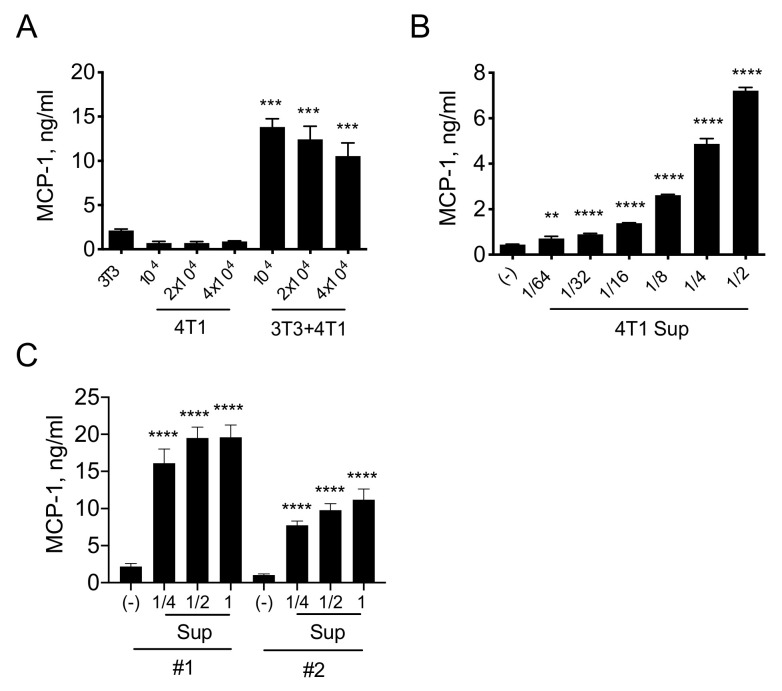
A product of 4T1 cells upregulates MCP-1 production by fibroblasts. (**A**) Ten thousand 3T3 cells, 1 × 10^4^, 2 × 10^4^ or 4 × 10^4^ 4T1 cells, or 1 × 10^4^ 3T3 cells with different numbers (1 × 10^4^, 2 × 10^4^, or 4 × 10^4^) of 4T1 cells were seeded into 24-well plates. After overnight incubation at 37 °C, the medium was replaced by 0.5 mL fresh medium. After a 4-day incubation at 37 °C, cell-free culture supernatants were collected, and the concentration of MCP-1 was measured by ELISA. Data are presented as mean ± SD. *** *p* < 0.0001, *n* = 3. Representative of three independent experiments with similar results. (**B**) Fifty thousand 3T3 cells were seeded into 24-well plates. After overnight incubation at 37 °C, the medium was replaced by 0.5 mL fresh medium containing different concentrations of 4T1-sup and incubated for 24 h at 37 °C. Cell-free culture supernatants were collected, and the concentration of MCP-1 was measured by ELISA. Data are presented as mean ± SD. ** *p* < 0.01, **** *p* < 0.00001. *n* = 3. Representative of three independent experiments with similar results. (**C**) Fifty thousand lung fibroblasts isolated from two normal BALB/c mice were seeded into 24-well plates. After overnight incubation at 37 °C, the medium was replaced by a fresh medium containing different concentrations of 4T1-sup. After a 24-h incubation, cell-free culture supernatants were collected, and the concentration of MCP-1 was measured by ELISA. Data are presented as mean ± SD. **** *p* < 0.00001, *n* = 3.

To examine the presence of crosstalk between 4T1 cells and fibroblasts that may contribute to the production of MCP-1 in 4T1 tumors, we cultured 4T1 cells and NIH/3T3 fibroblasts separately or together for 4 days in tissue culture plates and measured the concentration of MCP-1 in the supernatants. 3T3 cells constitutively released a basal level of MCP-1, and approximately 2.1 ng/mL of MCP-1 was detected in the 3T3 cell supernatant. 4T1 cells also constitutively released a low level of MCP-1 in the culture supernatant as we previously reported [12], and approximately 0.7 to 0.9 ng/mL of MCP-1 was detected (Figure 1A). When 3T3 cells were co-cultured with 4T1 cells, the concentration of MCP-1 in the culture supernatant increased by five to seven-fold. Since the capacity of 4T1 cells to produce MCP-1 is limited even after stimulation [12], this result strongly suggested that a product(s) of 4T1 cells activated 3T3 cells and upregulated MCP-1 production. To test this possibility, we cultured 3T3 cells with different concentrations of 4T1 cell-free culture supernatants (4T1-sup). As shown in Figure 1B, 4T1-sup dose-dependently increased the concentration of MCP-1 in the culture supernatant. Additionally, we used mouse primary fibroblasts isolated from the lungs of two normal BALB/c mice (#1 and #2) and cultured them in the presence of 4T1-sup. The concentration of MCP-1 in the culture supernatants markedly increased in response to 4T1-sup (Figure 1C). These results indicated that 4T1 cells release a factor that upregulates the production of MCP-1 by fibroblasts.

### 3.2. 4T1 Cells Produce and Release PDGFs

**Figure 2:**
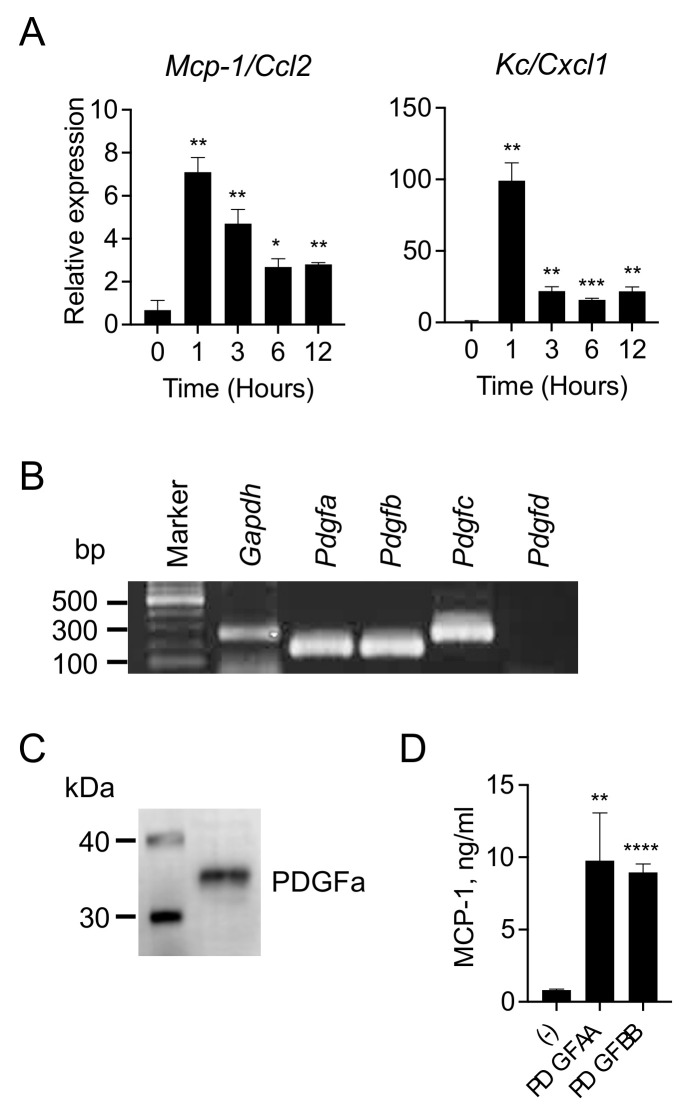
Increased MCP-1 production by fibroblasts in response to 4T1-sup was due to PDGFs. (**A**) Fifty thousand 3T3 cells were seeded into 24-well plates. After overnight incubation at 37 °C, the medium was replaced by 0.5 mL fresh medium containing 50% (*v*/*v*) of 4T1-sup and incubated for 1, 3, 6 or 12 h at 37 °C. Total RNA was isolated, and the expression of *Mcp-1* or *Kc/Cxcl1* mRNA was evaluated by qRT-PCR. Data are presented as mean ± SD. * *p* < 0.05, ** *p* < 0.01, *** *p* < 0.001, *n* = 3. Representative of two independent experiments with similar results. (**B**) Total RNA was isolated from 4T1 cells, and the expression of *Pdgfa*, *b*, *c*, and *d* mRNA was evaluated by RT-PCR. Representative of two independent experiments with similar results. (**C**) Ten μg of 4T1 cell lysate was loaded onto a polyacrylamide gel, and SDS-PAGE was performed. Proteins were transferred onto a membrane, and the presence of PDGF-A was examined by Western blotting. Representative of three independent experiments with similar results. (**D**) Fifty thousand 3T3 cells were seeded into 24-well plates. After overnight incubation at 37 °C, the medium was replaced by 0.5 mL fresh medium containing 100 ng/mL recombinant mouse PDGF-AA or PDGF-BB. After a 24-h incubation at 37 °C, cell-free culture supernatants were collected, and the concentration of MCP-1 was measured by ELISA. Data are presented as mean ± SD. ** *p* < 0.01, **** *p* < 0.00001, *n* = 3. Representative of four independent experiments with similar results.

MCP-1 is the product of the gene *JE*, one of the immediate early response genes induced in fibroblasts by PDGF [17,18,19], and several human breast cancer cell lines were found to produce PDGF in vitro [20], suggesting that MCP-1 production by fibroblasts in response to 4T1-sup may be due to PDGF. We first examined the kinetics of *Mcp-1* mRNA expression by 3T3 cells after stimulation with 4T1-sup. The expression of *Mcp-1* mRNA (Figure 2A, left panel) and *Kc* mRNA, another immediate early gene expressed by fibroblasts in response to PDGF (Figure 2A, right panel), was induced within 1 h after stimulation. These results supported the hypothesis that 4T1 cells produce PDGF(s) and activate fibroblasts to produce MCP-1.

There are four subtypes of PDGF, namely, PDGFa, b, c and d [21]. We examined the expression of these four PDGF subtypes by 4T1 cells by RT-PCR (Figure 2B). The expression of *Pdgf-a, b* and *c* mRNA, but not *d* mRNA, was readily detectable, and the production of PDGF-A protein was confirmed by Western blotting (Figure 2C). Finally, recombinant murine PDGF-AA and BB markedly upregulated MCP-1 production by 3T3 cells (Figure 2D). These results suggested that 4T1 cells upregulate MCP-1 production by fibroblasts via PDGF production.

### 3.3. The PDGF Receptor Inhibitor Crenolanib Inhibits 4T1 Cell-Induced MCP-1 Production by Fibroblasts

**Figure 3:**
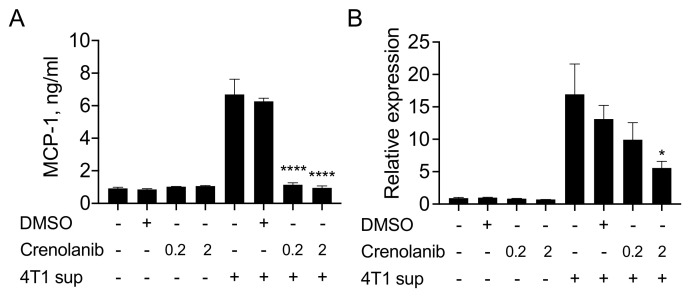
Pretreatment of 3T3 cells with crenolanib inhibited increased MCP-1 production induced by 4T1-sup. (**A**) Fifty thousand 3T3 cells were seeded into 24-well plates. After overnight incubation at 37 °C, the medium was replaced by a fresh medium containing DMSO or 0.2 or 2 μM crenolanib. After a 30-min incubation at 37 °C, the same volume of medium or 4T1-sup was added and incubated for an additional 24 h. Cell-free culture supernatants were collected, and the concentration of MCP-1 was measured by ELISA. Data are presented as mean ± SD. **** *p* < 0.00001, *n* = 3. Representative of three independent experiments with similar results. (**B**) Fifty thousand 3T3 cells were seeded into 24-well plates. After overnight incubation at 37 °C, the medium was replaced by a fresh medium containing DMSO or 0.2 or 2 μM crenolanib. After a 30-min incubation at 37 °C, the same volume of medium or 4T1-sup was added and incubated for an additional 24 h. Total RNA was isolated, and the expression of MCP-1 mRNA was evaluated by qRT-PCR. Data are presented as mean ± SD. * *p* < 0.05, *n* = 3. Representative of three experiments with similar results.

PDGFs bind to two closely related receptor tyrosine kinases, PDGFRA and PDGFRB [21]. To evaluate the contribution of PDGFs to the 4T1-sup-induced upregulation of MCP-1 production by fibroblasts, we blocked PDGFRA and PDGFRB by crenolanib, a highly potent inhibitor of these two receptors [22]. As shown in Figure 3A, Crenolanib showed no effect on the constitutive MCP-1 production by 3T3 cells but almost completely inhibited the increased MCP-1 production by 4T1-sup. Crenolanib also inhibited *Mcp-1* mRNA expression induced by 4T1-sup (Figure 3B). Crenolanib was initially developed as a highly selective PDGFR inhibitor [22] but later found to also inhibit FMS-like Tyrosine Kinase 3 (FLT3) [23,24]. Since FLT3 is expressed by hematopoietic cells but not by fibroblasts [25], the inhibitory effect of crenolanib in this experiment was considered via the blocking of PDGFRs. Crenolanib was also reported to inhibit cell proliferation [26,27], potentially influencing the level of MCP-1 in culture supernatants. However, *Mcp-1* mRNA expression by 3T3 cells peaked within 1 h of stimulation with 4T1-sup (Figure 2A), and crenolanib treatment had no effect on constitutive MCP-1 production or mRNA expression at the concentrations of 0.2 and 2 μM (Figure 3A,B). Taken together, these results supported the conclusion that increased MCP-1 production by fibroblasts in response to 4T1-sup was due to the activation of PDGF receptors by cancer cell-derived PDGFs.

### 3.4. PDGFR Antagonists Do Not Reduce the Level of MCP-1 in 4T1 Tumor-Bearing Mice

**Figure 4:**
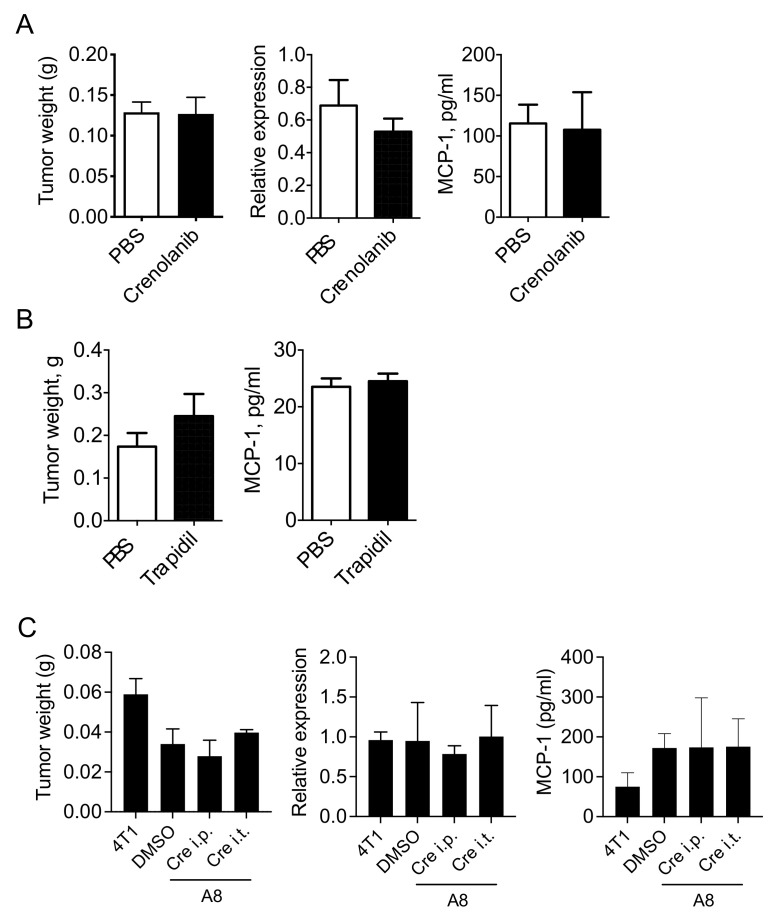
Treatment with trapidil or crenolanib had no effect on MCP-1 production or tumor growth. (**A**) One hundred thousand 4T1 cells were injected into a mammary pad of WT mice. Five days later, when tumors were clearly detectable, 100 μL of 10 μM crenolanib in PBS was intratumorally injected five times every 12 h. Mice were euthanized 4 h after the final injection, and the weight of the tumor (left panel) and the expression of *Mcp-1* mRNA in tumors (middle panel) and serum MCP-1 concentration (right panel) were measured. Data are presented as mean ± SD. *n* = 3 for each group. Representative of two independent experiments with similar results. (**B**) One hundred thousand 4T1 cells were injected into a mammary pad of WT BALB/c mice. Five days later, when tumors were clearly detectable, trapidil (20 mg/kg) in 100 μL PBS was intraperitoneally injected twice a day for 7 days. Mice were euthanized on the next day, and tumor weight and serum MCP-1 concentration were measured. Data are presented as mean ± SD. *n* = 3 for each group. Representative of two independent experiments with similar results. (**C**) One hundred thousand A8 cells were injected into a mammary pad of WT mice. Five days later, when tumors were clearly detectable, 500 μL of 10 μM crenolanib in PBS was injected i.p. twice a day for 3 days. To directly block PDGFRs in a tumor, 100 μL of 10 μM crenolanib in PBS was injected i.t. three times every 12 h. Mice were euthanized 12 h after the final injection, and the weight of the tumor (**left** panel), the expression of *Mcp-1* mRNA in tumors (**middle** panel) and serum MCP-1 concentration (**right** panel) were measured. Data are presented as mean ± SD. *n* = 3 for each group. Representative of two independent experiments with similar results.

We previously examined the kinetics of serum MCP-1 level in 4T1 tumor-bearing mice and found that the serum MCP-1 level significantly increased at 1 week, peaked at 3 weeks and then decreased at 4 weeks [12]. Since the sizes of tumors were significantly different between these time points, we attempted to evaluate the MCP-1 production rate at each time point by dividing the serum MCP-1 level by tumor size. Interestingly, MCP-1 production in tumors appeared highest at 1 week, and it gradually decreased (Figure S1). Therefore, we evaluated the contribution of the crosstalk between cancer cells and fibroblasts via PDGFs to the increased MCP-1 level in 4T1 tumor-bearing mice in the early phase of tumor development. As noted above, crenolanib inhibits not only PDGFRs but also FLT3 and reduces cell proliferation [23,24,25,26,27]. To minimize its systemic effects on both types of receptors and its inhibitory effect on cell proliferation, we injected 0.1 mL of 10 µM crenolanib directly into tumors five times every 12 h. Intratumoral injection has been used to deliver agents into tumors to kill cancer cells or to activate immune cells [28]. As shown in Figure 4A, there was no significant difference in tumor weight (left panel), *Mcp-1* mRNA expression in tumors (middle panel) or serum MCP-1 concentration (right panel) between the crenolanib-treated group and the control group.

We also injected 200 µg Trapidil twice per day for one week and collected sera and tumors. Trapidil was previously used to successfully block PDGFR in several animal disease models [29,30,31,32]. Trapidil treatment had no effects on tumor weight (Figure 4B, left panel) or serum MCP-1 concentration (Figure 4B, right panel).

We next transplanted GM-CSF-deficient, 4T1-derived clone A8 into WT mice and examined the effects of PDGFR inhibition (Figure 4C). Either i.p. or i.t. injection of crenolanib showed no effect on tumor weight (left panel), tumor *Mcp-1* mRNA expression (middle panel) or serum MCP-1 protein level (right panel) even in the absence of cancer cell-derived GM-CSF. These results strongly suggested that the PDGF-PDGFR pathway does not play a major role in the overall MCP-1 production in the 4T1 tumor.

### 3.5. MCP-1 Is Mainly Expressed by F4/80^+^ Macrophages, but Not by Fibroblasts, in 4T1 Tumors

**Figure 5:**
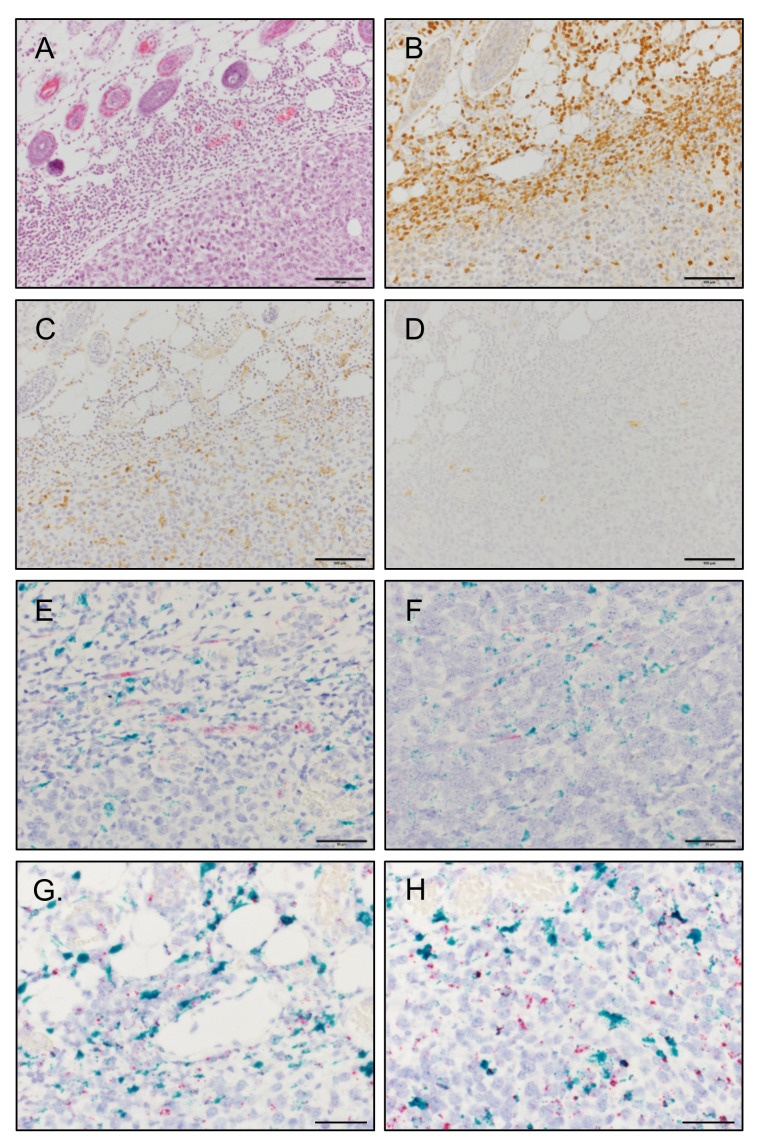
Histological examination and the detection of cells expressing *Mcp-1* mRNA in 5-day 4T1 tumors. One hundred thousand 4T1 cells in 100 μL PBS were injected into a mammary pad of WT female BALB/c mice. Five days after the injection, the mice were euthanized, and tumors were harvested. Consecutive paraffin sections from three individual tumors were examined by different staining and in situ hybridization. Representative photos are presented. (**A**) H&E staining, scale bar = 100 μm. (**B**) IHC with anti-Ly6G Ab, scale bar = 100 μm. (**C**) IHC with anti-F4/80 Ab, scale bar = 100 μm. (**D**) IHC with anti-αSMA, scale bar = 100 μm. (**E**) ISH. Green, *Mcp-1* mRNA; Red; *Act2* mRNA. Peritumoral area. Scale bar = 50 μm. (**F**) ISH. Green, *Mcp-1* mRNA; Red; *Act2* mRNA. Intratumoral area. Scale bar = 50 μm. (**G**) ISH. Green, *Mcp-1* mRNA; Red; *Adgre1* mRNA. Peritumoral area. Scale bar = 50 μm. (**H**) ISH. Green, *Mcp-1* mRNA; Red; *Adgre1* mRNA. Intratumoral area. Scale bar = 50 μm.

To further evaluate the contribution of fibroblasts to the MCP-1 production in 4T1 tumors, we histologically examined tumor tissues by immunohistochemistry (IHC). Five days after cancer cell inoculation, strong infiltration of leukocytes (Figure 5A), including Ly6G^+^ neutrophils (Figure 5B) and F4/80^+^ macrophages (Figure 5C), was observed. By contrast, only a small number of αSMA^+^ fibroblasts were found in 4T1 tumors (Figure 5D).

To determine MCP-1-producing cells in 4T1 tumors, we first used IHC; however, our attempts with multiple antibodies against murine MCP-1 were not successful. Therefore, we used in situ hybridization (ISH). By ISH, only a small number of *Act2* mRNA (red)-positive fibroblasts were found in peritumoral and intratumoral areas, and most of the *Mcp-1* mRNA (green)-positive cells were *Act2* mRNA (red)-negative (Figure 5E,F). *Mcp-1* mRNA (green) was mainly associated with *Adgre1*-positive macrophages (red) both in the peripheral area (Figure 5G) and inside tumors (Figure 5H).

**Figure 6:**
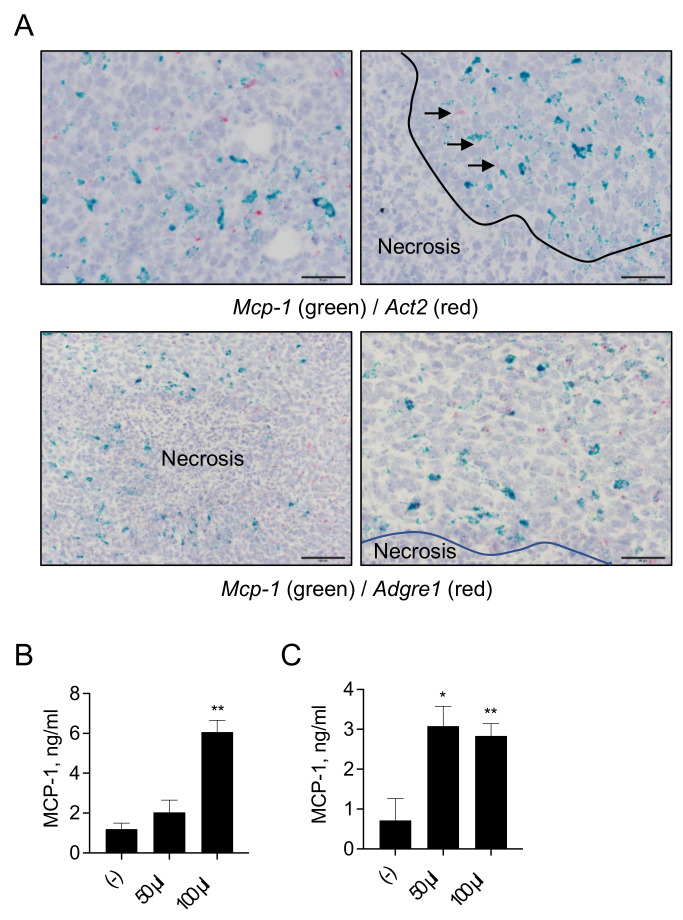
Detection of cells expressing *Mcp-1* mRNA in 14-day 4T1 tumors. One hundred thousand 4T1 cells in 100 μL PBS were injected into a mammary pad of WT female BALB/c mice. Fourteen days after the injection, the mice were euthanized, and tumors were harvested. (**A**) Consecutive paraffin sections from three individual tumors were examined for the expression of *Mcp-1* (green) and *Act2* (upper panels) or *Adgre1* (red) mRNA (lower panels) by ISH. Representative photos are presented. Scale bar = 50 μm. (**B**) One million peritoneal exudate macrophages were incubated for 24 h in the absence or presence of necrotic 4T1 cells, and the concentration of MCP-1 in the cell-free supernatants was measured by ELISA. Data are presented as mean ± SD. *n* = 3, ** *p* < 0.01. (**C**) One million peritoneal exudate macrophages were incubated for 24 h in the absence or presence of 50 or 100 μL of necrotic GM-CSF-deficient A8 cells, and the concentration of MCP-1 in the cell-free supernatants was measured by ELISA. Data are presented as mean ± SD. * *p* < 0.05, ** *p* < 0.01, *n* = 3. Representative of three independent experiments with similar results.

Fourteen days after cancer cell inoculation, the number of *Act2* mRNA (red)-positive fibroblasts remained low, and *Mcp-1* mRNA was not associated with *Act2* mRNA-positive fibroblasts (Figure 6A, upper panels). A large number of *Mcp-1* mRNA-positive cells were found around necrotic lesions, and the majority of *Mcp-1* mRNA-positive cells were also positive for *Adgre1* mRNA (Figure 6A, lower panels).

The effect of necrotic cancer cells on MCP-1 production by macrophages was examined in vitro. Necrotic 4T1 cells significantly upregulated MCP-1 production by mouse macrophages (Figure 6B). Necrotic GM-CSF-deficient A8 cells also upregulated MCP-1 production by macrophages (Figure 6C). These results indicated that macrophages are the major MCP-1 producing cells in 4T1 tumors and suggested that tumor necrosis contributes to the MCP-1 production by macrophages.

## 4. Discussion

Fibroblasts are a major cellular component of cancer stroma and secrete regulatory molecules, such as growth factors, cytokines and chemokines, which are important for cancer progression [14]. In the present study, we aimed to identify whether fibroblasts crosstalk with 4T1 breast cancer cells to produce MCP-1 in the TME [12]. The co-culturing of mouse fibroblasts with 4T1 cells or activation of fibroblasts with the culture supernatant of 4T1 cells (4T1-sup) resulted in elevated levels of MCP-1 production by fibroblasts. 4T1 cells expressed three PDGF isoforms, PDGF-a, b and c. Inhibition of PDGFs-PDGFR interaction by crenolanib almost completely inhibited 4T1-sup-induced MCP-1 production in vitro, but it had no effect in vivo. *Mcp-1* mRNA was mainly detected in *Adgre1*^+^ macrophages but not in *Act2*^+^ fibroblasts, likely CAFs. These results indicated that the crosstalk between 4T1 cells and fibroblasts indeed exists but is not significantly contributing to the MCP-1 production in the 4T1 breast cancer TME.

Human breast cancer cell line cells, such as MDA-MB-231 and MCF-7 cells, produce and secrete PDGF [20,33]. Although the exact role of PDGF in the development and progression of breast cancer remains unclear, PDGF is proposed as a potential target for breast cancer therapy [34]. PDGF is a potent mitogen for fibroblasts and an inducer of CAFs, which produce a variety of cytokines and chemokines, including MCP-1 [14,35]. In this study, we demonstrated that 4T1 cells have the capacity to upregulate MCP-1 production by fibroblasts via the secretion of PDGFs. However, inhibitors of PDGFRs had no effects on tumor *Mcp-1* mRNA expression or serum MCP-1 concentrations in tumor-bearing mice. Only a few *Act2*^+^ fibroblasts were present in 4T1 tumors on both days 5 and 14. Thus, PDGFs do not appear involved in the recruitment or proliferation of CAFs in the 4T1 tumor, and the lack of reduction in MCP-1 production by PDGFR inhibitors may be attributed to a low number of fibroblasts. Although CAFs might not be a major contributor to the MCP-1 production in the TME in an early phase of tumor development, as demonstrated here, it might be important in a later phase when an advanced tumor is established. It will also be interesting to examine the effects of long-term treatment by PDGFR inhibitors on the progression of 4T1 tumors, such as lung metastasis. Since the effectiveness of i.t. injection remains unproven, oral administration or the use of a subcutaneously implanted micropump that would provide a constant level of inhibitors in tumor microenvironments may be preferable. Additional studies are required to answer these questions.

Unlike 4T1 tumors, CAFs are the most abundant component of tumor stroma, especially in breast and pancreatic cancers in humans [35]; therefore, CAFs could be one of the major sources of MCP-1 in naturally arising tumors. The expression of MCP-1 in human invasive ductal breast cancer was previously studied by IHC [8,9,10,11]. MCP-1 was detected in cancer cells in roughly 50% of cases. Among stromal cells, TAMs were the major MCP-1-positive cells in all studies. Positive staining of endothelial cells or fibroblasts was also noted as minor cell populations [8,10]. Thus, despite the fact that CAFs are known to secrete MCP-1 in vitro, they may not significantly contribute to the production of MCP-1 in not only the 4T1 model but also naturally arising breast cancer. Additional studies using genetically engineered mouse models [36] or breast cancer cell lines, such as EO771 [37], would help us better understand the crosstalk between cancer cells and fibroblasts.

It was previously reported that conditioned media of human breast cancer cell lines or primary breast cancer cells upregulated MCP-1 production by human fibroblasts [38]. An increase in *MCP-1* gene promoter activity in response to the conditioned media was abrogated by a STAT3 antagonist. Several cytokines were present in the conditioned media as determined by human cytokine antibody arrays, but PDGFs were not among them. In our study, increased MCP-1 production by 4T1-sup was almost completely blocked by the PDGFR inhibitor crenolanib, indicating that PDGFs were the major 4T1 cell products that activated fibroblasts. Several factors are known to activate fibroblasts, and the availability of those factors may differ in each breast cancer TME. The identification of factors involved in the activation of fibroblasts in each cancer may be important to properly target the treatment of cancer patients.

We previously evaluated the contribution of myeloid and non-myeloid cells to the spontaneous lung metastasis of 4T1 cells by using BM chimera mice [12] and found that either cell type was sufficient to promote lung metastasis of 4T1 cells, suggesting that stromal cells, such as fibroblasts, produce a sufficient amount of MCP-1 for lung metastasis of 4T1 cells. In the present study, *Mcp-1* mRNA was mainly associated with *Adgre1*^+^ macrophages in tumors on both days 5 and 14 after the inoculation of cancer cells. To construct BM chimera, total-body irradiation and antibiotics treatment were required, and these procedures could affect the outcome of the experiment [39]. Studies using genetically engineered mouse models will be useful to obtain more definitive answers as to the source of MCP-1 in this cancer model.

*Mcp-1* mRNA-positive cells 14 days after cancer cell inoculation were mainly *Adgre1*^+^ macrophages infiltrating around necrotic lesions. This may suggest that those macrophages were activated by molecules released from dying or necrotic cancer cells. We tested this possibility by treating mouse macrophages with necrotic 4T1 cells or GM-CSF-deficient 4T1 clone cells and found that both cell types induced MCP-1 production. Necrotic cells release molecules, called danger-associated molecular patterns (DAMPs) or alarmins [40,41], among which HMGB1 is known to signal via toll-like receptor 4 [42]. Thus, the activation of macrophages with DAMPs is another mechanism for MCP-1 production in TMEs, but this mechanism may not function in an early stage of cancer development when necrosis of tumor cells is rare. It is likely that redundant, spatio-temporal activation mechanisms for macrophage activation are present in the 4T1 TME.

In conclusion, we have identified a novel crosstalk between 4T1 cells and fibroblasts for MCP-1 production in the 4T1 TME. However, it appears to be just one of many redundant crosstalks between 4T1 cells and stromal cells present in the 4T1 TME, and it is clear that blocking a single pathway is not sufficient to significantly reduce MCP-1 production. We believe that careful analysis of crosstalk between cancer cells and stromal cells and identification of molecules used in each tumor will help us to find better ways to treat patients with cancer, including breast cancer.

## Supplementary Materials

Supplementary materials can be found at https://www.mdpi.com/article/10.3390/cimb43030122/s1.
