# Supplementary material for: Crosstalk between Cancer Cells and Fibroblasts for the Production of Monocyte Chemoattractant Protein-1 in the Murine 4T1 Breast Cancer
Source: Curr Issues Mol Biol. 2021 Oct 22;43(3):1726–40. doi: 10.3390/cimb43030122 (PMC8928936; doi:10.3390/cimb43030122)
Supplement: Supplementary file 1 [file cimb-43-00122-s001.zip › cimb-1323670-supplementary.pdf]

Crosstalk between Cancer Cells and Fibroblasts for the Production of Monocyte Chemoattractant Protein-1 in the Murine 4T1 Breast Cancer

Mayu Imamura[1], Tiantian Li[1], Chunning Li[1], Masayoshi Fujisawa[1], Naofumi Mukaida[2], Akihiro Matsukawa[1] and Teizo Yoshimura[1,*]

[1]Department of Pathology and Experimental Medicine, Graduate School of Medicine, Dentistry and Pharmaceutical Sciences, Okayama University, 2-5-1 Shikata, Kita-ku, Okayama 700-8558, Japan.

[2]Division of Molecular Bioregulation, Cancer Research Institute, Kanazawa University, Kakuma-machi, Kanazawa 920-1192, Japan.

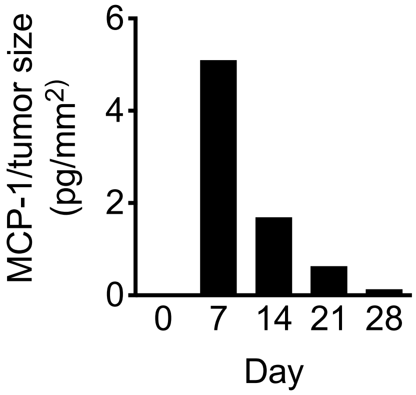

Figure S1. Kinetics of serum MCP-1 concentration per tumor size value in 4T1 tumor bearing mice. The values were calculated from the previously reported serum MCP-1 concentrations and tumor sizes after inoculation of 4T1 cells in BALB/c mice (Yoshimura T, et al., PLoS ONE 2013, 8, e58791).
